# Corneal Dendritic Cell Density Is Associated with Subbasal Nerve Plexus Features, Ocular Surface Disease Index, and Serum Vitamin D in Evaporative Dry Eye Disease

**DOI:** 10.1155/2016/4369750

**Published:** 2016-01-24

**Authors:** Rohit Shetty, Swaminathan Sethu, Rashmi Deshmukh, Kalyani Deshpande, Arkasubhra Ghosh, Aarti Agrawal, Rushad Shroff

**Affiliations:** ^1^Cornea and Refractive Services, Narayana Nethralaya, Bangalore 560 010, India; ^2^GROW Research Laboratory, Narayana Nethralaya Foundation, Bangalore 560 099, India

## Abstract

Dry eye disease (DED) has evolved into a major public health concern with ocular discomfort and pain being responsible for significant morbidity associated with DED. However, the etiopathological factors contributing to ocular pain associated with DED are not well understood. The current IVCM based study investigated the association between corneal dendritic cell density (DCD), corneal subbasal nerve plexus (SBNP) features, and serum vitamin D and symptoms of evaporative dry eye (EDE). The study included age and sex matched 52 EDE patients and 43 heathy controls. A significant increase in the OSDI scores (discomfort subscale) was observed between EDE (median, 20.8) and control (median, 4.2) cohorts (*P* < 0.001). Similarly, an increase in DCD was observed between EDE (median, 48.1 cells/mm^2^) patients and controls (median, 5.6 cells/mm^2^) (*P* < 0.001). A significant decrease in SBNP features (corneal nerve fiber length, fiber density, fiber width, total branch density, nerve branch density, and fiber area) was observed in EDE patients with OSDI score >23 (*P* < 0.05). A positive correlation was observed between DCD and OSDI discomfort subscale (*r* = 0.348; *P* < 0.0003) and SBNP features. An inverse correlation was observed between vitamin D and OSDI scores (*r* = −0.332; *P* = 0.0095) and DCD with dendritic processes (*r* = −0.322; *P* = 0.0122). The findings implicate DCD, SBNP features, and vitamin D with EDE symptoms.

## 1. Introduction

Dry eye disease (DED) is one of the common disorders of the eye with an estimated prevalence of 5.5%–33.7% worldwide [1]. Due to its high prevalence it is a public health concern with a significant economic burden. The hallmarks of DED include discomfort, visual disturbance, and tear film instability with potential damage to the ocular surface. It is accompanied by increased tear film osmolarity and inflammation of the ocular surface [2]. There has been widespread interest in understanding the disease and developing new treatment modalities for combating the ocular morbidity caused by it, especially the pain and discomfort associated with DED. Furthermore, in a subset of patients with DED the standard therapeutic strategies failed to alleviate the symptoms [3, 4]. Despite the knowledge available on the pathophysiological mechanisms of DED, there is a lack of substantial understanding with relevance to the etiopathology of the symptoms and their association with other in vivo clinical findings. The source of ocular discomfort or pain in DED cannot solely be explained by tear film metrics suggesting the role of other factors in causation of symptoms. Pain associated with dry eye has been described as neuropathic pain [5–7] and there have been emerging reports regarding dysfunctional ocular somatosensory nerves including the subbasal nerve plexus in ocular pain [8].

In vivo confocal microscopy (IVCM) has been extensively used to image the cornea at a cellular level both in ophthalmic clinical practice and in research. IVCM is used to study corneal diseases such as ectasias, keratitis, DED, and dystrophies [9]. Corneal nerves, epithelial cells, keratocytes, endothelial cells, and immune cells have been demonstrated on IVCM in different ocular and systemic diseases [8–11]. IVCM studies provide valuable insights into the etiology of DED and allow longitudinal imaging and quantification of cellular changes such as dendritic cells and subbasal nerve plexus morphology in the cornea of patients over time. Studies have demonstrated an increase in the corneal dendritic cell density in patients with DED [12–14]; however, its relevance to DED symptoms is yet to be investigated. Changes in corneal nerve morphology have been reported in keratoconus [15] and dry eye including those associated with systemic conditions such as chronic migraine, rheumatoid arthritis, chronic graft-versus-host disease, and Sjogrens syndrome [16–19].

Multiple etiologies including autoimmune diseases, aging, medications, refractive surgery, habits, diet, and environmental factors have been implicated in the pathophysiology of dry eye [20]. Recently, vitamin D, a fat-soluble prohormone with the ability to modulate calcium homeostasis and immune responses, has been associated with DED [21, 22]. Furthermore, there is also growing evidence regarding the potential role of vitamin D in chronic pain [23–25]. Similar to corneal nerve density and corneal nerve morphology, there is lack of evidence regarding the role of vitamin D and DED symptoms. Hence, in the current study the association between the severity of dry eye symptoms (pain and/or discomfort), corneal dendritic cell density, corneal subbasal nerve plexus features, and serum vitamin D was determined.

## 2. Materials and Methods

### 2.1. Study Population

The study was approved by the Ethics Committee of Narayana Nethralaya Hospital and was performed in accordance with the guidelines of the Declaration of Helsinki. Informed consent of study subjects was obtained at the time of enrollment. The subjects for this cross-sectional study to investigate the association between symptoms severity (pain or discomfort) and corneal dendritic cell density and corneal subbasal nerve plexus changes using in vivo confocal microscopy (IVCM) in evaporative dry eye (EDE) were selected from patients who presented to the Cornea Clinic at Narayana Nethralaya, Bangalore, India. A total of 52 patients (23 males and 29 females) who presented to our clinic with symptoms of EDE were included in the evaporative dry eye (EDE) group and 43 healthy volunteer subjects constituted the control group.

A thorough medical history was elicited to rule out any other ocular and systemic comorbidity, following which visual acuity, refraction, detailed slit-lamp and fundus evaluation, and DED investigations were performed. All the tests were performed under ambient conditions of temperature and humidity. A hanging drop of 1% fluorescein stain from fluorescein strip (ContaCare Ophthalmics and Diagnostics, India) was instilled in the cul-de-sac of the conjunctiva to measure the tear film break-up time (TBUT) in seconds and corneal and conjunctival epithelial staining, if present. Schirmer's test without anaesthetic was performed using sterile Schirmer's strips—Whatman filter paper (5 × 35-mm^2^, ContaCare Ophthalmics and Diagnostics, India). Schirmer strips were placed in the lower conjunctival sac at the junction of the lateral and middle thirds, without instilling anaesthesia. All patients were seated at rest with their eyes closed. Meibomian gland status was examined using infrared meibography (Oculus, Wetzlar, Germany) and was scored based on the loss of meibomian glands for each eyelid [26]. Patient ocular pain or discomfort was graded using ocular surface disease index (OSDI) questionnaire and the total OSDI scores were further classified into discomfort- and vision-related subscales [27]. Based on OSDI scores, the severity of symptoms can be grouped as normal (OSDI score of 0–12), mild (OSDI score of 13–22), moderate (OSDI score of 23–32), or severe (OSDI score of 33–100) [28]. Patients with OSDI scores indicating symptoms of dry eye, normal Schirmer's test values, and low TBUT were categorized as EDE. The control group included age matched healthy volunteers with Schirmer's test values > 10 mm and TBUT > 5 seconds and no symptoms of dry eye and other ocular conditions. Exclusion criteria include the use of contact lenses, the presence of drug allergy or ocular or systemic diseases with ocular manifestations such as Sjogren's syndrome, rheumatoid arthritis, and diabetes mellitus. Patients with disorders involving the lacrimal gland (congenital alacrimia, Steven-Johnson syndrome) and lid disorders including clinically evident meibomian gland dysfunction along with patients using topical medication were also excluded.

### 2.2. In Vivo Confocal Microscopy

IVCM imaging was performed using Rostock Corneal Module/Heidelberg Retina Tomograph ll (RCM/HRT ll, Heidelberg Engineering GmBH, Dossenheim, Germany) [7]. The device uses a diode laser of 670 nm wavelength. 0.5% proparacaine drops were used to anaesthetize the cornea before the procedure. Study subjects were asked to fixate on a distant target such as to enable examination of the central cornea. The central cornea was scanned in a single area at a desired depth. A drop of 0.5% moxifloxacin was instilled after the procedure. Image acquisition time was approximately 2 minutes per eye, and none of the subjects experienced any visual symptoms or corneal complications as a result of this examination. Both eyes were included for IVCM based investigations in the subjects of EDE cohort, whereas only one eye (right) was included for the control group.

### 2.3. Corneal Subbasal Nerve Plexus and Dendritic Cell Density Assessments

**Figure 1:**
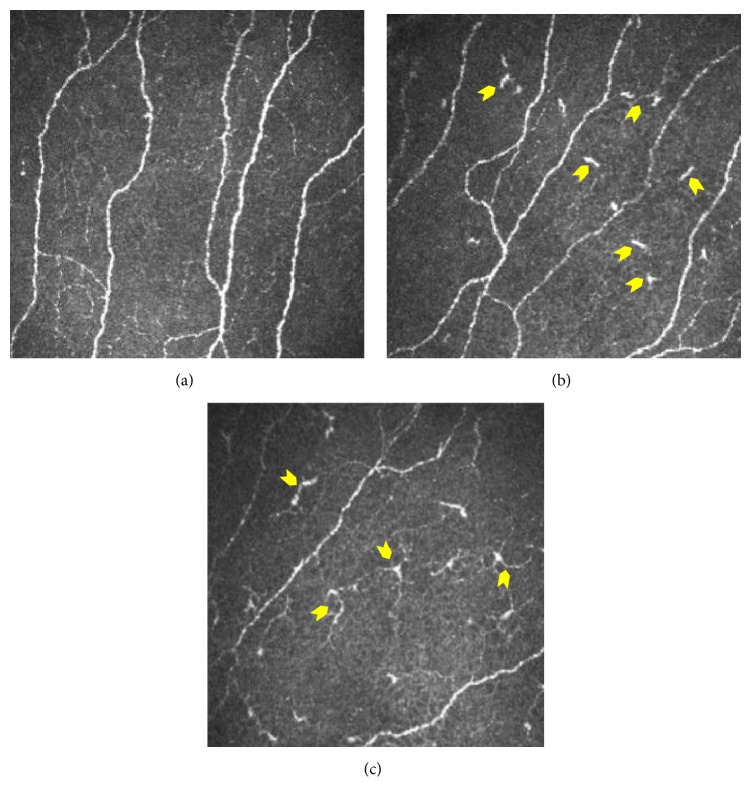
Dendritic cells at the level of subbasal nerve plexus in the cornea. Subbasal nerve plexus without dendritic cells (DCs) in a healthy control eye (a). DCs without dendritic process (b) and with dendritic processes (c) in dry eye patients. Panels shown are representative IVCM images with frame size 400 × 400 microns at a depth of 45 microns.

**Figure 2:**
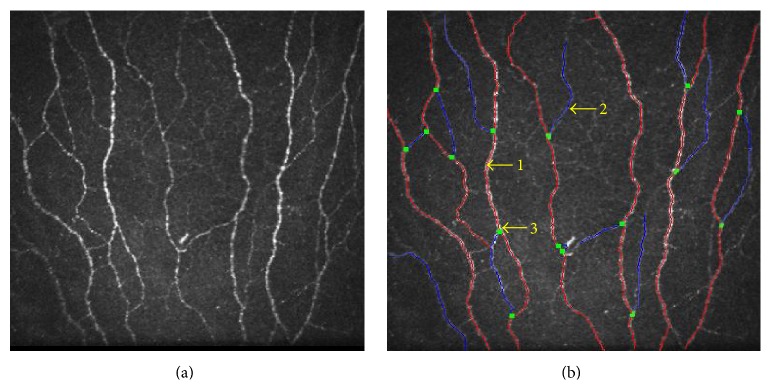
Morphological assessment of corneal subbasal nerve plexus. (a) Subbasal nerve plexus morphology as seen in a raw IVCM image. (b) Automated analysis of (a) using CC metrics software version 1.0 (University of Manchester, UK). “1” indicates the main nerve fibers highlighted in red. “2” denotes nerve fiber branches in blue. “3” as shown as green dots indicates branch points. Panels shown are representative IVCM images with frame size 400 × 400 microns at a depth of 50 microns.

An experienced masked observer selected five representative IVCM frames for corneal subbasal nerves and dendritic cells image based analyses. Images of the subbasal nerve plexus from the center of the cornea (Figure 1(a)) were assessed for each subject and for all the images the entire frame of 400 × 400 microns^2^ was used for analysis. Quantitative analyses of the nerve fibers were performed using Automatic CCMetrics software, version 1.0 (University of Manchester, UK) [29–33]. The parameters quantified as shown in Figure 1(b) include corneal nerve fiber density (CNFD), the total number of major nerves per square millimeter; corneal nerve fiber length (CNFL), the total length of all nerve fibers and branches (millimeters per square millimeter); corneal nerve branch density (CNBD), number of branches emanating from major nerve trunks per square millimeter; total branch density (CTBD), the total number of branch points per square millimeter; the nerve fiber area (CNFA) and the total nerve fiber area per square millimeter; and the corneal nerve fiber width (CNFW), the average nerve fiber width per square millimeter [29–31]. Dendritic cells (cells/mm^2^) were quantified using Cell Count software (Heidelberg Engineering GmbH) by identifying bright individual dendriform structures with cell bodies in each image at the level of basal epithelium or at subbasal nerve plexus [34]. Cells were included after assessment of two sides of the image for cells that overlapped with the edge of the frame. Bright cell bodies with and without dendritic processes or extensions were also identified (Figure 2). The images were analyzed by two blinded observers and the average of the values was used for statistical analysis.

### 2.4. Measurement of Serum Vitamin D

Serum was isolated from peripheral venous blood by using BD Vacutainer® Plus Plastic Serum Tubes (BD, New Jersey, USA). Total vitamin D—25 (OH) vitamin D levels—in the serum was measured by direct competitive chemiluminescent enzyme linked immunoassay (Euroimmun, Medizinische Labordiagnostika AG, Germany) that detects both 25 (OH) vitamins D_2_ and D_3_. The measurements were performed according to manufacturer's instructions.

### 2.5. Statistical Analysis

All statistical analyses were performed with MedCalc® version 12.5 (MedCalc Software bvba, Belgium) and GraphPad Prism 6.0 (GraphPad Software, Inc., La Jolla, CA, USA). Shapiro-Wilk normality test was done to check the distribution of the data set following which Spearman correlations analysis and Mann-Whitney test were used for further analyses. *P* < 0.05 was considered to be statistically significant. Data are represented as both mean ± SEM and median with range.

## 3. Results

Parameters such as TBUT, ocular surface disease index, corneal dendritic cell density (DCD), and corneal subbasal nerve plexus features were measured and analyzed in 43 (43 eyes) healthy controls and 52 (104 eyes) patients with EDE. The study subjects were age and gender matched. The ages between control (median 41 years; range 22–78 years) and EDE (median 44.5 years; range 19–73 years) cohort were not significantly different. Gender distribution (male/female) between the control and EDE cohort was 14M/29F and 23M/29F, respectively. TBUT was significantly lower in EDE subjects compared to controls (Table 1(a)). Total OSDI scores including discomfort- and vision-related OSDI subscales were observed to be significantly higher in the EDE cohort (Table 1(a)). An inverse correlation was observed between TBUT with total OSDI score (*r* = −0.32; *P* = 0.0009) and discomfort- (*r* = −0.354; *P* = 0.0002) and vision-related OSDI subscale (*r* = −0.197; *P* = 0.04).

**Figure 3:**
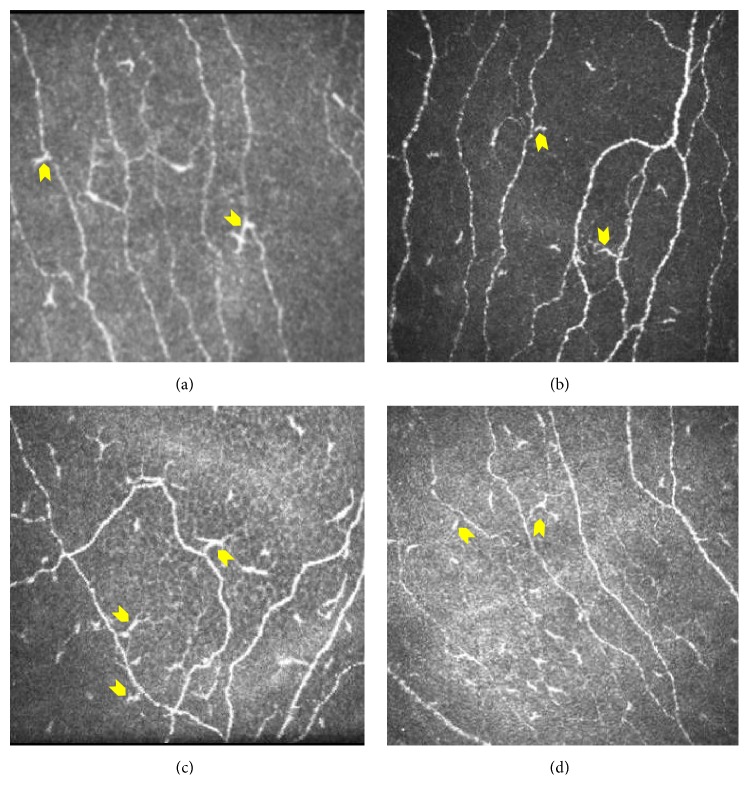
Anatomical localization of dendritic cells in relation to nerve fibers at the level of subbasal nerve plexus in the cornea of EDE patients. Yellow arrow indicates dendritic cells impinging or in close proximity to the nerve fibers. Panels shown are representative IVCM images from four EDE patients with frame size 400 × 400 microns at a depth of 50 microns.

IVCM investigations revealed the presence of corneal dendritic cells (DCs) in EDE (Figure 2). Image based analyses revealed a significant increase in corneal dendritic cell (DC) density and subsets (DCs with and without dendritic processes) in the eyes of EDE patients compared to controls (Table 1(a)). Analysis of subbasal nerve plexus features (as listed in Table 1(a)) from IVCM images revealed no significant difference between the study groups. However, significant decrease in nerve features such as nerve fiber length, branch points, and number of nerve branches was observed in EDE patients with moderate-to-severe OSDI scores (OSDI score > 23) compared to EDE patients with mild or normal OSDI scores (OSDI score < 23) and controls (Table 1(b)). In addition, number of major nerves and nerve fiber width were significantly lower in EDE patients with moderate-to-severe OSDI score compared to controls (Table 1(b)). OSDI score, specifically pain or discomfort-related subscale, exhibited a positive correlation with total corneal DC density, as well as density of DCs with and without dendritic process in EDE patients (Table 2). However, no correlation was observed between total OSDI score and vision-related OSDI subscale and corneal dendritic cell density in EDE patients (Table 2). Similarly, no association was observed between the various subbasal nerve plexus features and OSDI scores and subscale scores in EDE (Table 2). Nevertheless, a significant association between the corneal DC density (total, with and without dendritic process) and various subbasal nerve plexus features in EDE cohort was also observed as shown in Table 3. Furthermore, on many occasions a close proximity between DCs and the nerve fibers was observed in the EDE patients (Figure 3).

In addition, the relationship between serum vitamin D status and the various parameters studied was also investigated. The median serum vitamin D level in the EDE cohort (*n* = 30) was 16.4 ng/mL (range 5.8–61.9 ng/mL). Analyses demonstrated an inverse correlation between serum vitamin D level and OSDI scores—total and discomfort- and vision-related subscales (Table 4). The density of corneal DCs with dendritic process revealed an inverse correlation with serum vitamin D in EDE patients (Table 4). However, no associations were observed between serum vitamin D and total corneal DC density, DCs without dendritic process, or subbasal nerve plexus features in the EDE cohort (Table 4).

## 4. Discussion

The persistence of ocular pain and discomfort in a subset of patients with DED following standard therapeutic strategies as well as the lack of tear film metrics to predict this population poses a major challenge in the management of DED. It is therefore imperative to identify diagnostic modalities that can accurately predict patients whose symptoms may not resolve with conventional therapy or may require additional dietary or environmental interventions along with topical therapy to ensure a favourable prognosis. IVCM used to study architecture of the cornea in dry eye and other ocular conditions can provide additional predictive information such as corneal DCD and SBNP features which are altered in DED. In our study, we observed a significant association between OSDI scores especially the discomfort subscale with corneal DCD. Despite the absence of correlation between the decreased SBNP features and OSDI in EDE patients, we did observe a significant decrease in a subset of EDE patients with moderate-to-severe OSDI. Furthermore, a significant different correlation was also observed between DCD and SBNP features. These observations implicate the changes in DCD and SBNP morphology with symptoms observed in EDE.

Reports on subbasal nerve plexus have revealed conflicting findings. Benítez-Del-Castillo et al. showed a significant decrease in the nerve density in patients with dry eye [19] which is similar to what we have observed in the current study. Similar observations were made in other studies on dry eye with relation to chronic migraine and chronic graft-versus-host disease [16, 17]. However, Hoçal et al. reported no difference in subbasal nerve density whereas Zhang et al. demonstrated increased corneal nerve density in patients with dry eye [35, 36]. The variations observed could be due to the influence of the underlying disease. A recent report demonstrated a positive correlation between changes in subbasal nerve morphology features (decrease in CNFL, CNFD, and CNBD) and pain in patients with diabetic neuropathy [37]. Similary, in our current study we have also observed a significant decrease in various nerve features in EDE patients with moderate-to-severe symptoms, thus suggesting the use of corneal nerve morphological features as a predictor of the presence of pain in EDE patients. The mechanistic basis of this association is necessary to validate this observation. Neuropathic pain such as dysesthesias and hyperalgesia in dry eye patients can be due to either peripheral sensitization of neurons or damage to free nerve endings that interdigitate between superficial epithelial cells and are exposed to environmental and/or inflammatory stimuli. The presence of inflammation has also been found to directly and indirectly affect the structure and function of peripheral nerves resulting in altered nociception [38]. On the other hand excited nerve fibers can secrete neuropeptides which in turn trigger a neurogenic inflammatory response.

The role of dendritic cells in modulation of nociception and pain has been previously studied [39, 40]. Dendritic cells play a role in immunomodulation and in antigen presentation and may influence pain pathways through their effect on T helper cells. Studies have described a possible role for corneal dendritic cells in the etiopathogenesis of dry eye, keratoconjunctivitis sicca, and corneal allograft rejection [41, 42]. Inflammatory pathologies show an increase in the number of dendritic cells in the cornea [43]. Lin et al. demonstrated an increase in dendritic cells in the anterior stroma along with activation of epithelial dendritic cells as documented by the presence of more dendrites in the center of the cornea [44]. In our study the significant increase in the corneal dendritic cells observed in EDE patients was found to have positive association with the OSDI discomfort-related subscale scores and not vision-related OSDI scores. The current study also reports a differential association between corneal dendritic cells and SBNP features in EDE. Tuisku et al. demonstrated altered stromal corneal nerves and the presence of increased antigen presenting cells in patients with dry eye. They proposed that these changes were responsible for dysesthesia experienced by the patient in dry eye disease. In their study, however, they did not describe association between the dendritic cell density and changes in the corneal nerves [45]. We propose that an increase in inflammatory cells and the associated changes in subbasal nerve plexus may be responsible for ocular discomfort experienced by patients in our cohort. Furthermore, an increase in the number of dendritic cells in close proximity to the subbasal nerves was observed in patients with severe symptoms. Whether DC-mediated inflammatory or physical irritation of the nerve or changes in nerve physiology are responsible for pain in these patients needs to be determined. Therefore, this incidental observation warrants further investigation.

Vitamin D and its role in the etiopathogenesis of dry eye disease have been the subject of many recent research publications. Studies have demonstrated the association of vitamin D deficiency with DED [21, 22, 25]. In the current study we observed a strong inverse correlation between the OSDI scores and vitamin D levels in the EDE cohort. Earlier reports have suggested vitamin D deficiency to be associated with neuralgia and chronic pain [23, 25]. Vitamin D exhibits anti-inflammatory and immunoregulatory properties and its deficiency results in inflammatory or immune mediated dryness of the eyes. Apart from its effect on tear film indices, the impact of vitamin D levels on ocular pain or discomfort has not been explored in detail. Vitamin D can influence the severity of symptoms by modulating nociception by regulating nerve homeostasis and inflammatory responses. The exact mechanism linking vitamin D to pain remains elusive; however several theories have been put forward. Serotonin which can perpetuate chronic pain response was found to be high in patients with DED [46] and vitamin D is known to affect serotonin synthesis [47] indicating a role of vitamin D in nociception. Studies have shown that vitamin D decreases production of nitric oxide, a nociceptive neurotransmitter, thereby modulating pain [48, 49]. Vitamin D and its agonists have been found to inhibit maturation and induce tolerance in dendritic cells resulting in the arrest of inflammatory processes [50]. Lower vitamin D levels were associated with an increase in DCs with dendritic processes (mature phenotyple) in our cohort which supports the current understanding regarding the immunomodulatory role of vitamin D on DCs. Vitamin D also modulates the expression of various inflammatory cytokines in various cells, including corneal epithelial cells [51] substantiating the anti-inflammatory/immunomodulatory functions of vitamin D. Our observations suggest that the increased corneal dendritic cells density and its potential effect on the subbasal nerve plexus features could contribute to the severity of symptoms in EDE. Furthermore, low vitamin D level can result in severe symptoms by directly influencing nociception on nerve fibers and/or indirectly by lack of negative regulation on DCs activation/migration and inflammatory responses. However, a more detailed mechanistic investigation is necessary to validate it.

## Figures and Tables

**(a):** 

	Control (*n* = 43; 43 eyes)			EDE (*n* = 52; 104 eyes)			*P* value
	Mean	Median	Range	Mean	Median	Range	
OSDI							
Total	15.0 ± 1.5	14.9	0–38.5	28.1 ± 2.5	20.8	2.1–68.8	0.0005^*∗*^
OSDI—discomfort	4.5 ± 0.5	4.2	0–12.5	32.6 ± 3.2	20.8	4.2–83.3	<0.0001^*∗*^
OSDI—vision	6.4 ± 0.8	8.3	0–20.8	23.5 ± 2.4	20.8	0–70.8	<0.0001^*∗*^
TBUT	10.7 ± 0.3	10	7–15	7.0 ± 0.2	7	1–12	<0.0001^*∗*^
Dendritic cells							
Total cells (cells/mm^2^)	9.1 ± 1.3	5.6	0–36.8	52.9 ± 4.0	48.1	7–261.2	<0.0001^*∗*^
DCs with dendrites (cells/mm^2^)	1.0 ± 0.2	0	0–4	13.0 ± 1.2	8.5	0–54.6	<0.0001^*∗*^
DCs without dendrites (cells/mm^2^)	8.1 ± 1.2	5.2	0–35.4	39.9 ± 3.2	29.8	7–235	<0.0001^*∗*^
Subbasal nerve plexus							
CNFL (length in mm/mm^2^)	17.0 ± 0.4	17.7	7.1–22.3	16.5 ± 0.3	16.27	8.09–22.98	ns
CNFD (major nerves/mm^2^)	28.6 ± 0.8	29.7	12.5–40	27.2 ± 0.6	27.5	5–43.75	ns
CNFW (average nerve fiber width/mm^2^)	0.0211 ± 0.0002	0.0209	0.0186–0.0254	0.0211 ± 0.00009	0.021	0.0184–0.0237	ns
CTBD (branch points/mm^2^)	58.4 ± 3.2	58.75	3.7–103.7	56.8 ± 2.5	52.5	15–123.7	ns
CNBD (number of branches/mm^2^)	40.9 ± 2.3	42.35	1.2–83.7	39.0 ± 1.7	37.5	3.75–81.24	ns
CNFA (total nerve fiber area/mm^2^)	0.0066 ± 0.0002	0.0066	0.002–0.0111	0.0068 ± 0001	0.00665	0.0034–0.0134	ns

EDE: evaporative dry eye; OSDI: ocular surface disease index; TBUT: tear break-up time; DCs: dendritic cells; CNFL: corneal nerve fiber length; CNFD: corneal nerve fiber density; CNFW: corneal nerve fiber width; CTBD: corneal total branch density; CNBD: corneal nerve branch density; CNFA: corneal nerve fiber area; ns: not statistically significant; ^*∗*^ *P* value compared to controls (Mann-Whitney test).

**(b):** 

	EDE (OSDI score < 23) *n* = 58 eyes			*P* value	EDE (OSDI score > 23) *n* = 46 eyes			*P* value
	Mean	Median	Range		Mean	Median	Range	
Subbasal nerve plexus								
CNFL (length in mm/mm^2^)	16.9 ± 0.3	17.7	7.1–22.3	ns^*∗*^	15.9 ± 0.4	15.8	8.09–22.3	0.0165^*∗*^; 0.04^#^
CNFD (major nerves/mm^2^)	27.9 ± 0.7	28.7	15–42.5	ns^*∗*^	26.2 ± 1.0	27.5	5–43.75	0.0447^*∗*^; ns^***#***^
CNFW (average nerve fiber width/mm^2^)	0.0210 ± 0.0001	0.021	0.0193–0.0233	ns^*∗*^	0.0211 ± 0.0001	0.021	0.0184–0.0237	0.6107^*∗*^; ns^#^
CTBD (branch points/mm^2^)	61.5 ± 3.5	59.3	15–123.7	ns^*∗*^	50.8 ± 3.4	48.7	15–111.2	0.0398^*∗*^; 0.04^#^
CNBD (number of branches/mm^2^)	42.7 ± 2.3	41.2	11.2–81.2	ns^*∗*^	34.3 ± 2.6	32.5	3.75–78.7	0.0208^*∗*^; 0.01^#^
CNFA (total nerve fiber area/mm^2^)	0.0071 ± 0.0002	0.0069	0.0035–0.0134	ns^*∗*^	0.0064 ± 0002	0.0063	0.0034–0.0119	0.4098^*∗*^; ns^#^

EDE: evaporative dry eye; OSDI: ocular surface disease index; CNFL: corneal nerve fiber length; CNFD: corneal nerve fiber density; CNFW: corneal nerve fiber width; CTBD: corneal total branch density; CNBD: corneal nerve branch density; CNFA: corneal nerve fiber area; ns: not statistically significant; ^*∗*^ *P* value compared to controls (Mann-Whitney test); ^#^ *P* value compared to EDE—OSDI score < 23 (Mann-Whitney test).

**Table 2:** Correlation of OSDI scores with corneal dendritic cell density and corneal subbasal nerve plexus features in EDE patients.

	OSDI—discomfort		OSDI—vision		OSDI—total	
	*r*	*P* value	*r*	*P* value	*r*	*P* value
Dendritic cells						
Total cells (cells/mm^2^)	**0.348**	**0.0003**	−0.104	0.2925	0.161	0.1028
DCs with dendrites (cells/mm^2^)	**0.274**	**0.0048**	−0.039	0.6937	0.126	0.2016
DCs without dendrites (cells/mm^2^)	**0.347**	**0.0003**	−0.118	0.2335	0.162	0.0999
Subbasal nerve plexus				
CNFL (length in mm/mm^2^)	0.148	0.1342	0.079	0.427	0.157	0.112
CNFD (major nerves/mm^2^)	0.097	0.3292	0.167	0.0897	0.153	0.1215
CNFW (average nerve fiber width/mm^2^)	0.009	0.9247	0.009	0.9259	0.027	0.7824
CTBD (branch points/mm^2^)	0.124	0.2101	0.009	0.9249	0.102	0.3027
CNBD (number of branches/mm^2^)	0.094	0.3411	0.073	0.459	0.117	0.2352
CNFA (total nerve fiber area/mm^2^)	0.18	0.067	−0.103	0.2991	0.07	0.4787

OSDI: ocular surface disease index; DCs: dendritic cells; CNFL: corneal nerve fiber length; CNFD: corneal nerve fiber density; CNFW: corneal nerve fiber width; CTBD: corneal total branch density; CNBD: corneal nerve branch density; CNFA: corneal nerve fiber area; *r*: Spearman correlation coefficient.

**Table 3:** Correlation between corneal dendritic cell density and corneal subbasal nerve plexus features in EDE patients.

	Dendritic cells					
Subbasal nerve plexus	DCs with dendrites		DCs without dendrites		Total DCs	
	*r*	*P* value	*r*	*P* value	*r*	*P* value
CNFL (length in mm/mm^2^)	0.004	0.9705	0.036	0.7147	0.028	0.7752
CNFD (major nerves/mm^2^)	**−0.223**	**0.0229**	−0.177	0.0723	**−0.213**	**0.03**
CNFW (average nerve fiber width/mm^2^)	**0.277**	**0.0044**	0.169	0.0855	**0.228**	**0.0201**
CTBD (branch points/mm^2^)	**0.213**	**0.0299**	**0.219**	**0.0254**	**0.244**	**0.0124**
CNBD (number of branches/mm^2^)	0.066	0.505	0.109	0.272	0.108	0.2737
CNFA (total nerve fiber area/mm^2^)	**0.419**	**<0.0001**	**0.427**	**<0.0001**	**0.463**	**<0.0001**

OSDI: ocular surface disease index; DCs: dendritic cells; CNFL: corneal nerve fiber length; CNFD: corneal nerve fiber density; CNFW: corneal nerve fiber width; CTBD: corneal total branch density; CNBD: corneal nerve branch density; CNFA: corneal nerve fiber area; *r*: Spearman correlation coefficient.

**Table 4:** Association of serum vitamin D with OSDI score, corneal dendritic cell density, and corneal subbasal nerve plexus features in EDE patients.

	Serum vitamin D level	
	*r*	*P* value
OSDI score		
OSDI—total	**−0.332**	**0.0095**
OSDI—discomfort	**−0.375**	**0.0032**
OSDI—vision	**−0.289**	**0.025**
Dendritic cells		
Total cells (cells/mm^2^)	−0.184	0.1589
DCs with dendrites (cells/mm^2^)	**−0.322**	**0.0122**
DCs without dendrites (cells/mm^2^)	−0.099	0.45
Subbasal nerve plexus		
CNFL (length in mm/mm^2^)	−0.004	0.9749
CNFD (major nerves/mm^2^)	0.037	0.777
CNFW (average nerve fiber width/mm^2^)	−0.083	0.5267
CTBD (branch points/mm^2^)	−0.094	0.4742
CNBD (number of branches/mm^2^)	−0.007	0.96
CNFA (total nerve fiber area/mm^2^)	−0.011	0.9334

OSDI: ocular surface disease index; DCs: dendritic cells; CNFL: corneal nerve fiber length; CNFD: corneal nerve fiber density; CNFW: corneal nerve fiber width; CTBD: corneal total branch density; CNBD: corneal nerve branch density; CNFA: corneal nerve fiber area; *r* – Spearman correlation coefficient.
